# Supplementary material for: The Light-Controlled Release of 2-fluoro-l-fucose, an Inhibitor of the Root Cell Elongation, from a nitrobenzyl-caged Derivative
Source: Int J Mol Sci. 2023 Jan 28;24(3):2533. doi: 10.3390/ijms24032533 (PMC9916816; doi:10.3390/ijms24032533)
Supplement: Supplementary file 1 [file ijms-24-02533-s001.zip › ijms-2089347-supplementary.pdf]

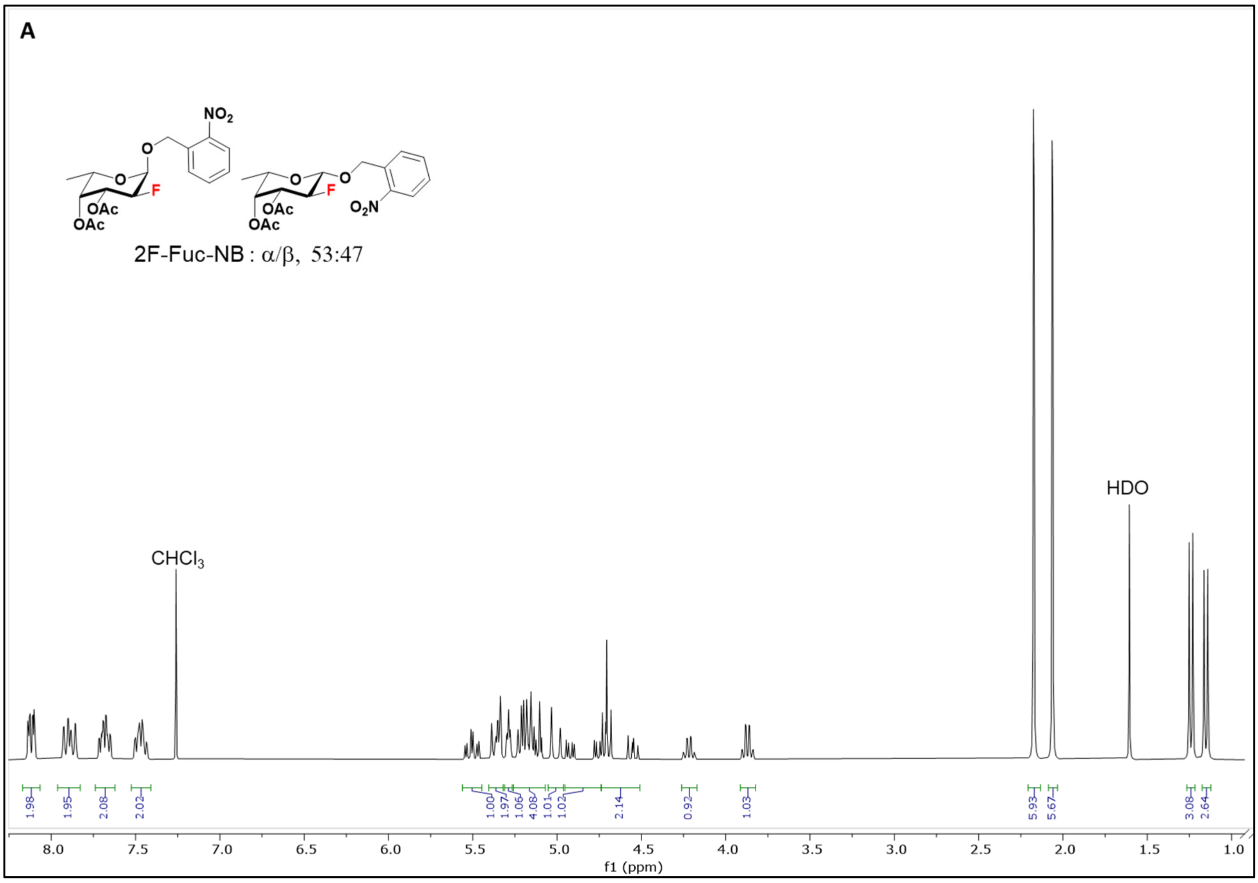
A
NO2
O
O
F
OAc
OAc
O2N
2F-Fuc-NB : α/β, 53:47
CHCl3
HDO
1.98
1.95
2.08
2.02
1.00
1.97
1.06
4.08
1.01
1.02
2.14
0.92
1.03
5.93
5.67
3.08
2.64
8.0 7.5 7.0 6.5 6.0 5.5 5.0 4.5 4.0 3.5 3.0 2.5 2.0 1.5 1.0
f1 (ppm)

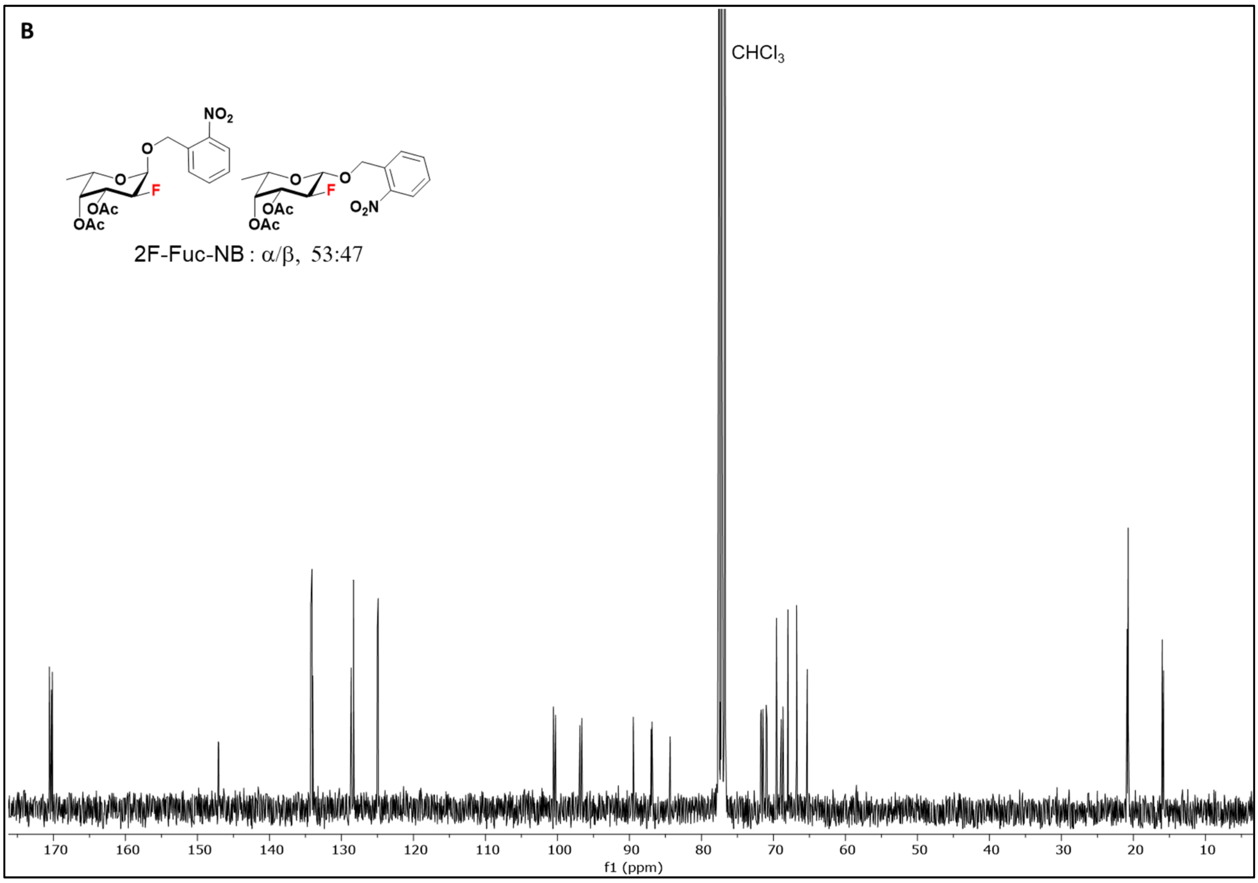
B
NO2
O
O
F
OAc
OAc
O2N
2F-Fuc-NB : α/β, 53:47
CHCl3
170 160 150 140 130 120 110 100 90 80 70 60 50 40 30 20 10
f1 (ppm)

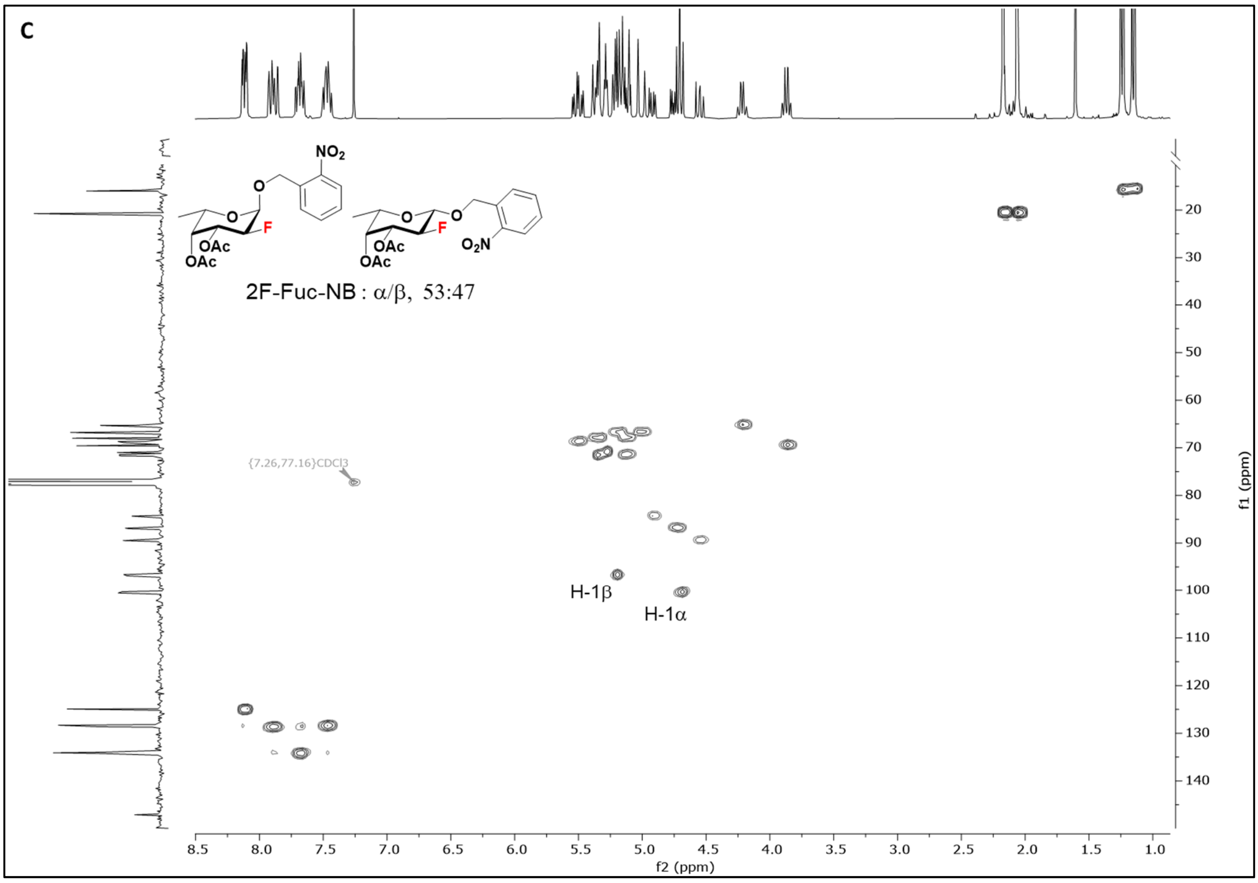

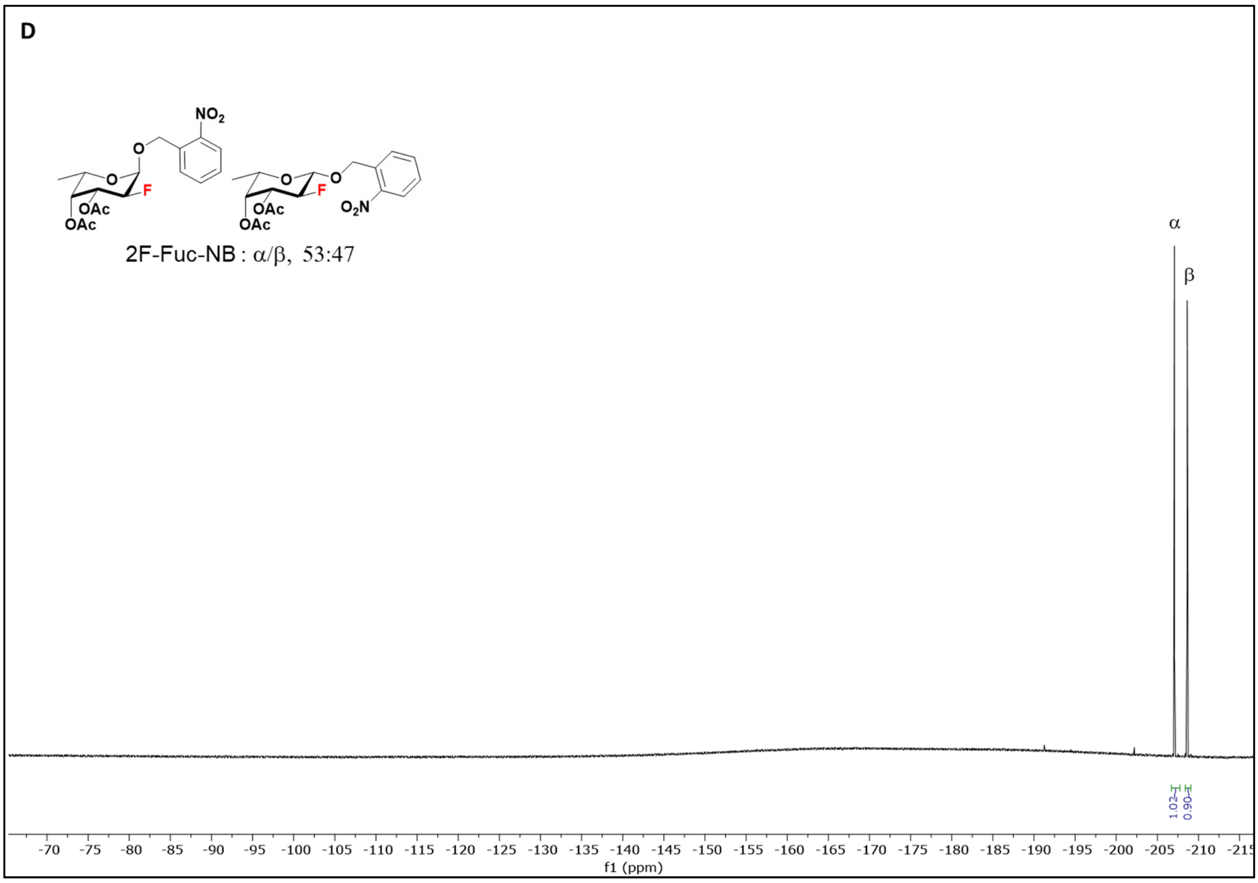

Figure S1. ^{1}H (A), ^{13}C (B), 2D HSQC (C) and (^{1}H uncoupled)-^{19}F (D) NMR spectra of the mixture of α and β anomers (α/β: 53/47) of 3,4-di-*O*-acetyl-1-*ortho*-nitrobenzyl-2-fluoro-L-fucose (2F-Fuc-NB). Solvent: $CDCl_3$.

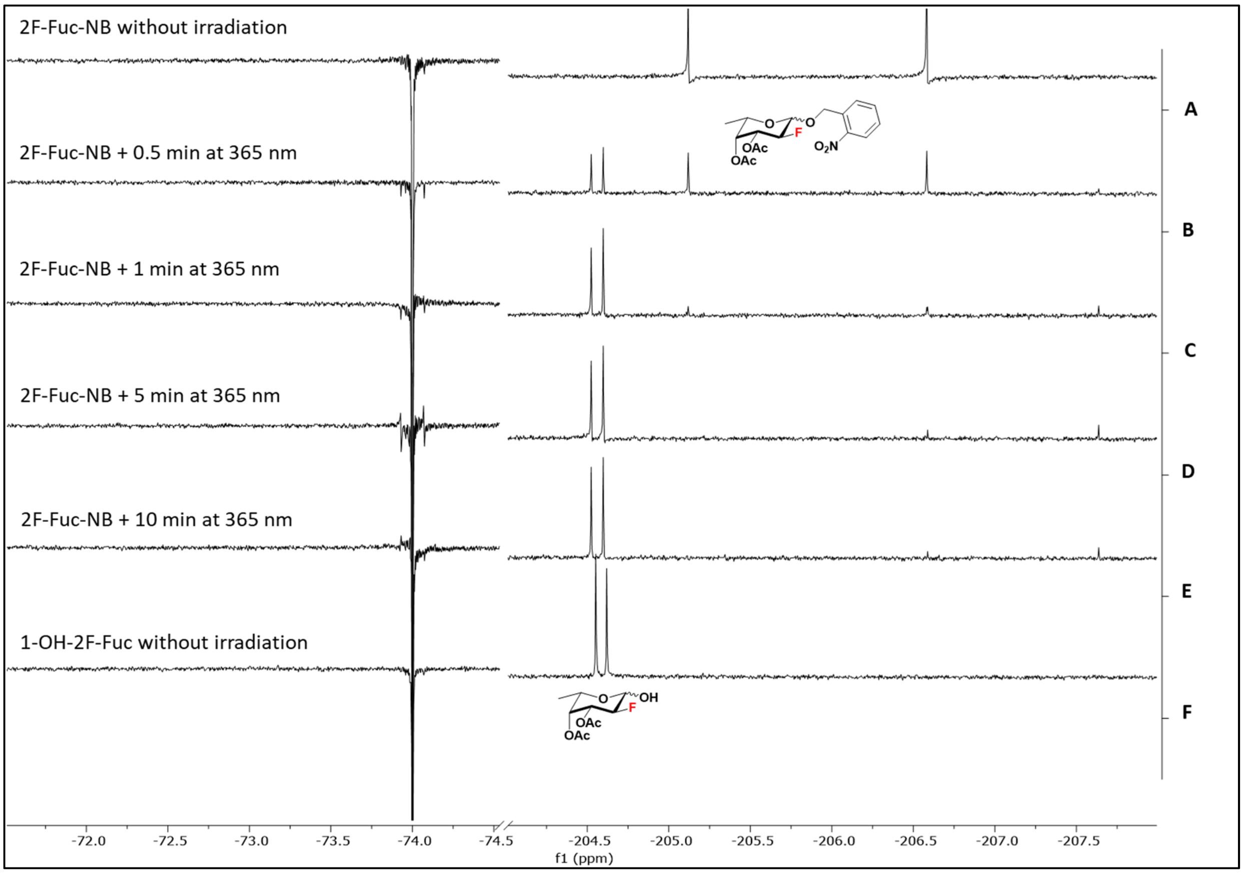

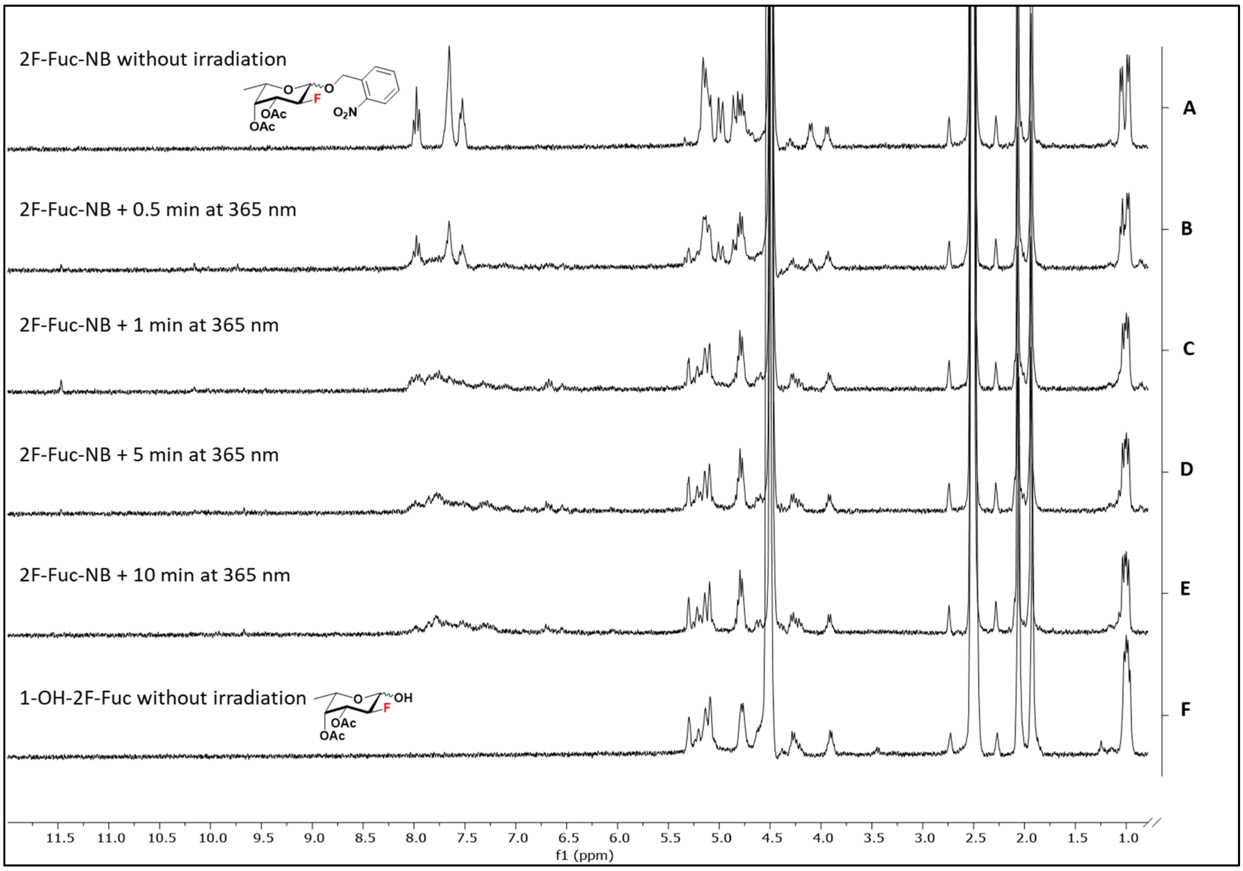

Figure S2. Monitoring by proton-uncoupled ^{19}F and ^{1}H NMR of 2F-Fuc-NB (2 mM in D_2O/DMSO-d_6, 1:1, v/v) before and after photoirradiation at 365 nm over 10 min. t= 0, 0.5, 1, 5 and 10 min (A-E). 3,4-di-*O*-acetyl-1-hydroxy-2-fluoro-L-fucose (F). Reference: hexafluoropropan-2-ol at – 74 ppm.

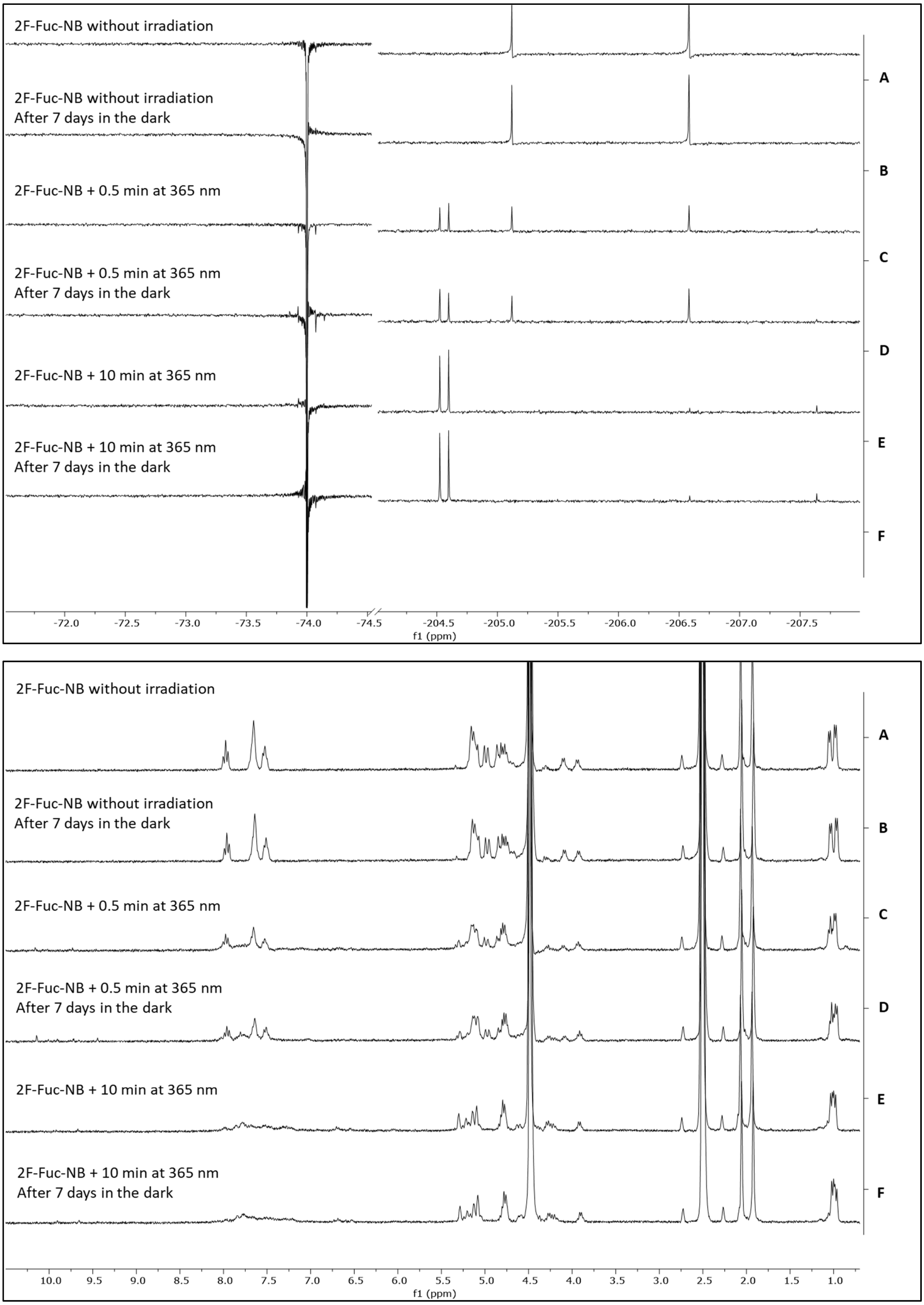

Figure S3. Monitoring of stability over 7 days in the dark by proton-uncoupled ^{19}F and ^{1}H NMR of 2F-Fuc-NB (2 mM in D_2O/DMSO-d_6, 1:1, v/v) after photoirradiation at 365 nm over 10 min. t= 0, 0.5, and 10 min (A, C, E). t= 0, 0.5, and 10 min after 7 days in the dark (B, D, F). Reference: hexafluoropropan-2-ol at – 74 ppm.

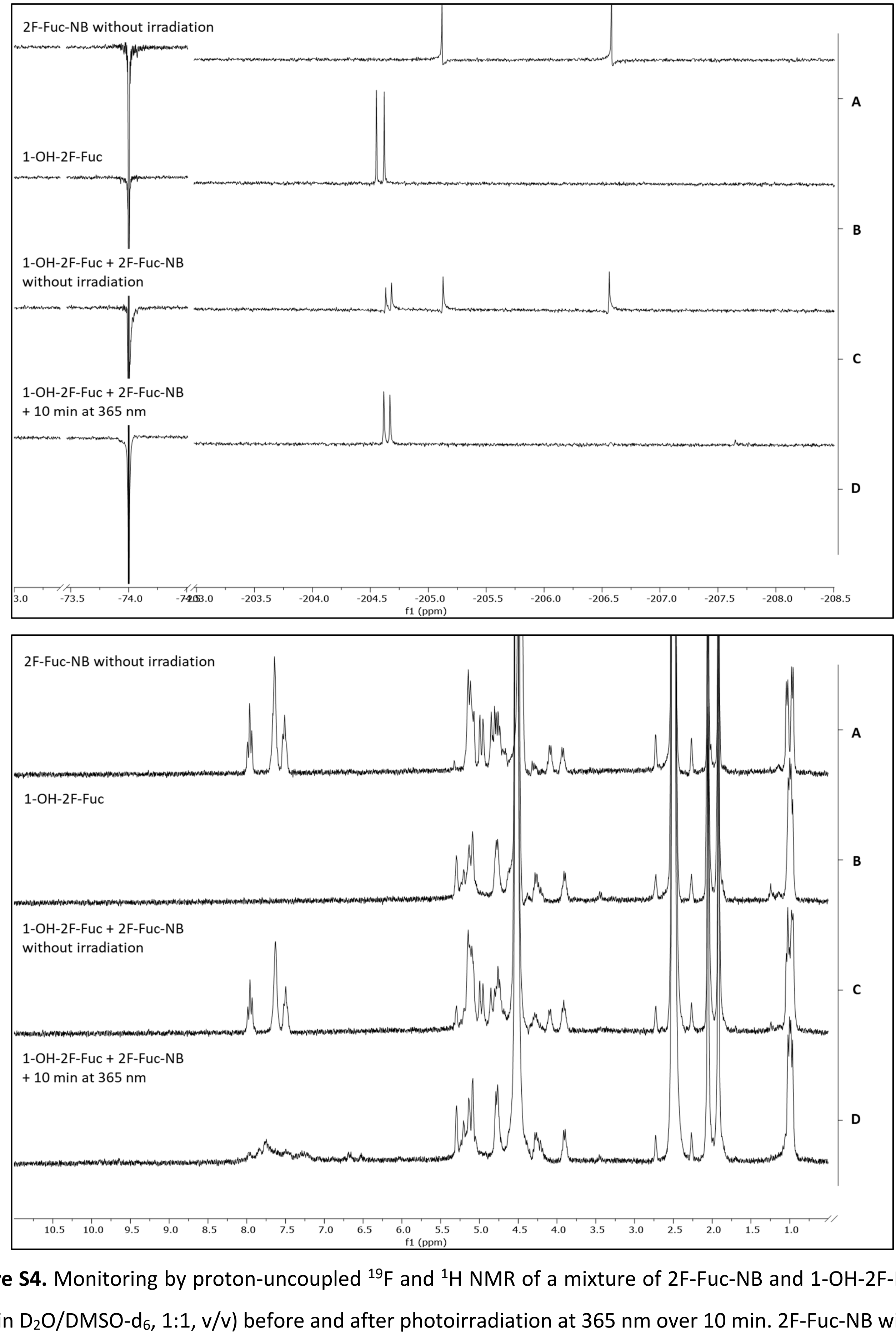

Figure S4. Monitoring by proton-uncoupled ^{19}F and ^{1}H NMR of a mixture of 2F-Fuc-NB and 1-OH-2F-Fuc (2 mM in D_2O/DMSO-d_6, 1:1, v/v) before and after photoirradiation at 365 nm over 10 min. 2F-Fuc-NB without irradiation (A), 1-OH-2F-Fuc (B), mixture of 2F-Fuc-NB and 1-OH-2F-Fuc without irradiation (C), mixture of 2F-Fuc-NB and 1-OH-2F-Fuc after 10 min at 365 nm (D). Reference: hexafluoropropan-2-ol at – 74 ppm.
